# Adverse Effects of Airborne Heavy Metals on Human Skin Cells: Insights From Sebum Analysis of Indoor and Outdoor Workers

**DOI:** 10.1155/jt/7925949

**Published:** 2026-08-31

**Authors:** Hye-Won Na, Nari Cha, Eun Joo Kim, Sanghun Kim, Won-Seok Park, Hyoung-June Kim

**Affiliations:** ^1^ Division of Advanced Beauty Science, Research and Innovation Center, Amorepacific, Yongin, South Korea

**Keywords:** air pollution, heavy metals, inflammation, oxidative stress, skin barrier dysfunction

## Abstract

Heavy metals in particulate matter (PM) contribute to oxidative stress, DNA damage, and inflammatory responses in skin cells. This study aimed to investigate the toxic effects of airborne heavy metals on human sebocytes, keratinocytes, and ex vivo skin. To this end, we collected sebum samples from indoor (*n* = 62) and outdoor (*n* = 40) workers in Shanghai and quantified the concentrations of 24 metals using inductively coupled plasma mass spectrometry (ICP‐MS). Outdoor workers exhibited higher levels of cobalt (0.0004 vs. 0.39 mg/kg, 924.7‐fold), arsenic (0.11 vs. 14.15 mg/kg, 134.7‐fold), barium (0.70 vs. 20.24 mg/kg, 28.8‐fold), and manganese (0.86 vs. 14.93 mg/kg, 17.3‐fold) than indoor workers. Sebum‐mimicking metal mixtures (SMMs), formulated to reflect the specific heavy metal profiles identified in the workers′ sebum, were applied to human epidermal keratinocytes and sebocytes. Outdoor SMMs significantly reduced cell viability, increased lipid peroxidation, and induced DNA damage, as evidenced by the phosphorylation of histone H2AX (γH2AX) foci formation. Real‐time quantitative reverse‐transcription polymerase chain reaction (RT‐qPCR) analysis revealed upregulated *IL1A, IL1B, TNFA, IL6,* and *PTGS2* mRNA, primarily after treatment with outdoor SMMs. Further activation of the NF‐κB and MAPK signaling pathways was also identified. This was corroborated in human scalp skin models, in which exposure to outdoor SMMs increased the expression of proinflammatory cytokines, including interleukin‐1β (IL‐1β) and IL‐6, and decreased the expression of keratin 10. These findings highlight the detrimental effects of environmental heavy metals on skin homeostasis, providing a scientific basis for the development of antipollution skincare formulations.

## 1. Introduction

Air pollution has emerged as a critical environmental and public health challenge in recent decades, mainly because of rapid industrialization and expanding urban populations worldwide. As cities grow, the concentration of atmospheric pollutants, particularly in densely populated areas, increases markedly, threatening human health and the environment [1–4]. Extensive epidemiological research has established associations between chronic air pollution exposure and various systemic health outcomes, including respiratory diseases, cardiovascular disorders, and increased mortality [5–8]. Emerging evidence further indicates that air pollution has a significant detrimental impact on skin health [9–11]. The skin serves as the primary interface between the human body and the external environment, making it a primary target organ for atmospheric pollutants through direct contact and systemic circulation following inhalation or ingestion [9, 12]. Fine particulate matter (PM) with aerodynamic diameters below 2.5 μm (PM2.5) is of particular concern in air pollution due to its ability to penetrate biological tissues [10, 13]. PM can adhere to skin surfaces and penetrate hair follicles and pores [14], while ultrafine particles < 4 nm can traverse intact skin barriers [15]. The chemical composition of PM includes heavy metals that demonstrate redox activity and promote reactive oxygen species (ROS) generation, resulting in oxidative damage to cellular proteins, lipids, and DNA [13, 16].

Among the atmospheric contaminants, heavy metals such as lead (Pb), cadmium (Cd), mercury (Hg), arsenic (As), nickel (Ni), and chromium (Cr) have garnered attention owing to their persistence in the environment and potential adverse health effects even at relatively low concentrations [17–19]. In contrast to organic pollutants, which may degrade, heavy metals tend to accumulate in environmental matrices and biological systems, leading to prolonged exposure risks for humans [20, 21]. Airborne heavy metals cause skin toxicity through several interconnected pathways, including oxidative stress from ROS production, weakened antioxidant defenses, and inflammation [16, 22]. They bind to protein sulfhydryl groups, induce apoptosis, interfere with DNA repair, and may lead to genomic instability and carcinogenic transformation [22].

Specifically, PM penetrates skin pores, and the associated heavy metals interact with sebum, releasing proinflammatory mediators, including interleukin (IL)‐1β, IL‐6, and tumor necrosis factor‐alpha (TNF‐α), which act on surrounding keratinocytes and sebocytes through paracrine signaling mechanisms [23, 24]. This inflammatory cascade can result in barrier dysfunction, accelerated aging, and the development of various dermatological conditions, including eczema, acne, and psoriasis [25–27].

Sebum is a lipid‐rich secretion produced by sebaceous glands that plays a role in skin physiology by maintaining moisture, providing antimicrobial properties, and forming a barrier against environmental insults [28]. The lipophilic nature of sebum suggests its potential to absorb lipophilic pollutants, making it a candidate biomarker for assessing environmental heavy‐metal exposure. While sebum composition is known to change in response to various environmental conditions, the specific relationship between environmental heavy metal exposure and sebum composition remains underexplored. Previous studies, including our transcriptome‐based analysis, have demonstrated that PM2.5 exposure induces oxidative stress, inflammatory signaling, and lipid metabolic dysregulation in human sebocytes [29].

However, these studies primarily focused on PM as a bulk pollutant and did not address the contribution of sebum‐associated heavy metals under real‐world occupational exposure conditions.

Occupational exposure provides a biologically relevant and practical framework for investigating real‐world differences in environmental pollutant burden. Workers engaged in outdoor activities are continuously exposed to ambient airborne pollutants, including heavy metals, whereas indoor workers experience relatively reduced exposure due to enclosed environments and air filtration systems. Therefore, comparing indoor and outdoor workers enables a clear stratification of exposure levels, allowing us to directly link environmental metal burden with its accumulation in sebum and subsequent biological effects on keratinocytes and sebocytes. Importantly, this design allows us to model real‐world exposure scenarios and establish a direct exposure–response relationship, which cannot be achieved using conventional in vitro exposure systems alone. In addition, Shanghai was selected as the study area due to its high population density and well‐documented levels of air pollution, making it an appropriate real‐world model for studying environmentally relevant heavy metal exposure. Accordingly, this population‐based design directly supports the objective of this study to investigate the relationship between environmental metal exposure, sebum accumulation, and skin cellular responses under real‐world conditions. Therefore, this study aims to compare sebum heavy metal concentrations between indoor and outdoor workers to establish exposure–response relationships and identify populations more susceptible to pollution‐related skin disorders.

Despite the growing recognition of the impact of air pollution on skin health, significant knowledge gaps remain regarding the specific mechanisms by which heavy metals affect specific skin cells. In particular, limited research has directly compared the toxicity of heavy metals in sebocytes and keratinocytes, which play essential roles in skin physiology. Furthermore, while real‐world PM exposure involves multiple heavy metals simultaneously, most studies have focused on single‐metal exposures, leaving their combined toxicological effects largely unknown.

Understanding the cellular and molecular mechanisms underlying heavy metal toxicity in skin cells is fundamental for advancing effective prevention and treatment strategies for pollution‐related skin disorders [16, 22]. The identification of sensitive biomarkers and clarification of key pathways implicated in metal‐induced skin damage can inform occupational health guidelines and therapeutic interventions, protecting high‐risk groups from the adverse effects of environmental heavy metal exposure [16, 22].

Therefore, in this study, we aimed to address existing knowledge gaps by evaluating the toxicological effects of airborne heavy metals on human skin cells, with an emphasis on the distinct responses observed in sebocytes and keratinocytes. By examining sebum samples from indoor and outdoor workers to reflect real‐world exposure scenarios and subsequently assessing cellular responses to sebum‐mimicking metal mixtures (SMMs), which are synthetic solutions designed to reflect the exact heavy metal concentrations found in the sebum samples, this study provides important insights into the occupational health risks associated with heavy metal exposure and supports evidence‐based strategies to protect skin health in polluted environments.

## 2. Materials and Methods

### 2.1. Collection of Sebum Samples and Analysis of Metal Contents

A total of 102 healthy volunteers were enrolled in this cross‐sectional study: 62 indoor workers and 40 outdoor workers. The cohort comprised female and male participants aged 21–60 years, with an overall mean age of 41.84 ± 11.23 years. To ensure a rigorous contrast in environmental exposure, participants were categorized based on their actual daily outdoor exposure time, rather than their occupational titles. Specifically, the indoor group consisted of individuals with less than 2 h of daily outdoor exposure (including commuting), whereas the outdoor group included those with 6 h or more. Participants were required to have observable sebum in the nasal and paranasal areas to be included in the study. The average age was 39.99 ± 11.52 years for the indoor group and 43.72 ± 10.65 years for the outdoor group. The composition of fine dust, including heavy metals and other harmful constituents, was determined.

Sebum samples were collected from each participant using sterile fine‐tipped tweezers under controlled conditions. All assessments and sample collections were conducted in a climate‐controlled laboratory maintained at a temperature of 22 ± 2°C and relative humidity of 45 ± 5%. To ensure that the facial skin was genuinely exposed to ambient air, the participants were required to refrain from wearing face masks during their shifts. Furthermore, to prevent external contamination, they were strictly prohibited from applying any cosmetics or sunscreen to the facial areas prior to sampling. Prior to collection, the skin surface was gently cleansed with a cotton swab moistened with ethanol to remove superficial contaminants, including dust and cosmetic residues, without disturbing the sebaceous secretion.

Sebum was extracted by carefully grasping the superficial lipid layer with tweezers and lifting it away from the skin, minimizing contact with adjacent tissue. This method was selected for its specificity in removing sebum while limiting exogenous contamination and keratinous debris. Each specimen was immediately transferred to a preweighed, metal‐free microcentrifuge tube to verify that a minimum of 2 mg of sebum was collected from each participant for accurate analysis. To prevent cross‐contamination, a new set of sterilized tweezers was used for each participant. The samples were sealed and stored at −20°C until further processing to preserve the integrity of the lipid and metal content. All procedures were performed using gloved hands in a laminar flow hood to maintain sterility and ensure sample quality.

The total metal concentrations in the sebum samples were quantified using inductively coupled plasma mass spectrometry (ICP‐MS), following standard protocols for trace metal assessment in biological matrices [30, 31]. Briefly, each sample underwent acid digestion with ultrapure nitric acid and was then diluted with deionized water. The digested specimens were subsequently analyzed for the selected elements.

### 2.2. Cell Culture and Viability Assay

Human sebocytes (Celprogen, Torrance, CA, USA) and normal human epidermal keratinocytes (NHEK; Lonza, Basel, Switzerland) were cultured in Human Sebocyte Complete Growth Media with Serum (Celprogen) and KBM Gold basal medium supplemented with KGM Gold SingleQuots (Lonza), respectively. Cells were incubated at 37°C in a humidified atmosphere containing 5% CO_2_, and the medium was changed every 2 days.

Cell viability was assessed using the Cell Counting Kit‐8 (Dojindo Laboratories, Kumamoto, Japan) according to the manufacturer’s instructions. SMMs were formulated based on the heavy metal concentrations detected in sebum samples from indoor and outdoor workers. These solutions were serially diluted up to 100‐fold and subsequently applied to sebocytes and keratinocytes for viability testing.

### 2.3. Chemicals

Sodium meta‐arsenite (NaAsO_2_), cobalt(II) chloride hexahydrate (CoCl_2_·6H_2_O), barium chloride (BaCl_2_), manganese(II) chloride tetrahydrate (MnCl_2_·4H_2_O), nickel(II) chloride (NiCl_2_), lithium chloride (LiCl), lead(II) nitrate (Pb(NO_3_)_2_), potassium chromate (K_2_CrO_4_), and cadmium chloride hemipentahydrate (CdCl_2_·2.5H_2_O) were dissolved in distilled water and sterilized by filtration through a 0.22‐μm syringe to prepare 10‐mM stock solutions. Germanium(IV) oxide (GeO_2_) was prepared at the required concentration in the culture media, and iron(II) sulfate heptahydrate (FeSO_4_·7H_2_O) was dissolved in distilled water to 100 mM. All metal compounds were obtained from Sigma‐Aldrich (St. Louis, MO, USA).

### 2.4. Real‐Time Quantitative Reverse‐Transcription Polymerase Chain Reaction (RT‐qPCR)

Following exposure to SMMs at 37°C for 24 h, total RNA was isolated from the keratinocytes using TRIzol reagent (Ambion, Carlsbad, CA, USA) according to the manufacturer’s guidelines. RNA concentration and quality were assessed using a NanoDrop spectrophotometer (Thermo Fisher Scientific, Waltham, MA, USA). cDNA was synthesized from 1 μg of RNA using the RevertAid First Strand cDNA Synthesis Kit (Invitrogen, Carlsbad, CA, USA) according to the manufacturer’s protocol. RT‐qPCR analyses were performed using 1 μL of cDNA, TaqMan Universal Master Mix II, and specific TaqMan probes on a 7500 Fast Real‐Time PCR system (Applied Biosystems, Foster City, CA, USA). The TaqMan probes (Applied Biosystems) were specific for *TNFA* (Hs00174128_m1), *IL1A* (Hs00174092_m1), *IL1B* (Hs01555410_m1), *PTGS2* (Hs00153133_m1), *IL6* (Hs00174131_m1), keratin 10 (*KRT10*; Hs00166289_m1), and ribosomal protein L13a (*RPL13A*; Hs04194366_g1).

### 2.5. Measurement of Lipid Peroxidation

Sebocytes were cultured on collagen I‐coated glass coverslips (Thermo Scientific). Lipid peroxidation was assessed 24 h after treatment with SMMs, with BODIPY 581/591 C11 (2 μM; Thermo Fisher Scientific) added for the final 30 min. Hoechst 33342 (Thermo Fisher Scientific) stained nuclei. After three washes with PBS, the cells were imaged using a confocal microscope (LSM890; Zeiss, Oberkochen, Germany).

### 2.6. Quantification of Phosphorylation of Histone H2AX (γ‐H2AX)

Sebocytes were seeded, treated with SMMs for 24 h, washed with PBS, and fixed with 4% paraformaldehyde. For immunofluorescence, the cells were stained with a mouse anti‐γ‐H2AX antibody (Merck Millipore, Darmstadt, Germany) and Alexa Fluor 488‐conjugated goat anti‐mouse IgG (Invitrogen). Subsequently, they were mounted with a mounting medium containing 4′,6‐diamidino‐2‐phenylindole (DAPI, P36962; Invitrogen) for nuclear staining. Imaging was performed using a confocal microscope (LSM890; Zeiss).

### 2.7. Western Blotting of Cell Lysates

Cells were lysed with RIPA lysis buffer and resuspended in sodium dodecyl sulfate (SDS) sample buffer containing a reducing buffer. The lysates were resolved using sodium dodecyl sulfate‐polyacrylamide gel electrophoresis (SDS‐PAGE) and transferred to a polyvinylidene difluoride membrane. The membrane was blocked with blocking buffer (#37536; Thermo Fisher Scientific) and incubated with primary antibodies. The antibodies used included anti‐phospho‐NF‐κB p65 (Ser536; #3033), anti‐NF‐κB p65 (#8242), anti‐phospho p38 MAPK (Thr180/Tyr182; #4511), p38 MAPK (#8690), anti‐γH2AX (#9718), and anti‐GAPDH (#2118), all from Cell Signaling Technology (Beverly, MA, USA). All primary antibodies were used at a 1:1000 dilution. Detection was performed using horseradish peroxidase‐conjugated anti‐mouse IgG (#31430) or goat anti‐rabbit secondary antibodies (#31460) and SuperSignal West Chemiluminescence Reagent (Thermo Fisher Scientific).

### 2.8. Human Scalp Skin Tissue and Immunohistochemistry

Human scalp skin tissue was purchased from Biopredic International (Rennes, France) in compliance with French Law L.1245‐2 CSP and maintained in a 2‐week culture medium without animal components (Biopredic International). The tissue samples were exposed to SMMs at 1:12.5 and 1:50 dilutions for 6 days, followed by fixation in 10% formalin for paraffin embedding and sectioning. Paraffin‐embedded tissue sections were stained with the following antibodies: IL‐1β (Abcam; Waltham, MA, USA), IL‐6 (Abcam), and KRT10 (Santa Cruz Biotechnology; Paso Robles, CA, USA).

### 2.9. Statistical Analysis

Data are presented as mean ± standard deviation (SD) from three independent experiments. Statistical analyses were performed using SPSS Statistics (Version 24.0). Comparisons among multiple groups were conducted using one‐way analysis of variance (ANOVA) followed by Tukey’s HSD post hoc test. For comparisons between two groups, Student′s *t*‐test was used. A *p* value < 0.05 was considered statistically significant.

## 3. Results

### 3.1. Metal Concentrations in Sebum

**FIGURE 1:**
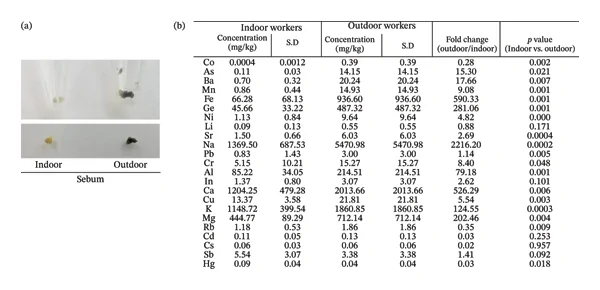
Comparison of sebum appearance and heavy metal composition between indoor and outdoor workers. (a) Representative images of sebum samples collected from indoor and outdoor workers. (b) Heavy metal analysis of sebum samples from indoor and outdoor workers. Concentrations of trace and heavy metals were quantified (mg/kg) and are presented as mean ± standard deviation (SD). Fold change values represent the ratio of the average concentrations in outdoor workers to those in indoor workers. Statistical comparisons between groups were conducted using Student′s *t*‐test.

Sebum samples were collected from both outdoor and indoor workers (Figure 1a), and the metal compositions were analyzed (Figure 1b). Sebum samples from outdoor workers contained significantly higher concentrations of several heavy metals than those from indoor workers (Figure 1b). Specifically, the Co levels measured 0.39 ± 0.28 mg/kg in outdoor samples and 0.0004 ± 0.0012 mg/kg in indoor samples, representing a 924.7‐fold increase (*p* < 0.01). Similarly, concentrations in outdoor sebum samples were 134.7‐fold higher for As (14.15 ± 15.30 mg/kg vs. 0.11 ± 0.03 mg/kg, *p* < 0.05), 28.8‐fold for barium (Ba) (20.24 ± 17.66 mg/kg vs. 0.7 ± 0.32 mg/kg, *p* < 0.01), 3.6‐fold for Pb, and 3.0‐fold for Cr compared to indoor samples.

SMMs, designated as the indoor and outdoor SMMs, were prepared based on the concentrations of 11 metallic components: eight metals (Co, As, Ba, Mn, Fe, Ge, Ni, and Li) with more than a 5‐fold concentration difference, as well as Pb, Cd, and Cr, recognized for their toxicity.

### 3.2. Cell Viability in NHEKs and Human Sebocytes

**FIGURE 2:**
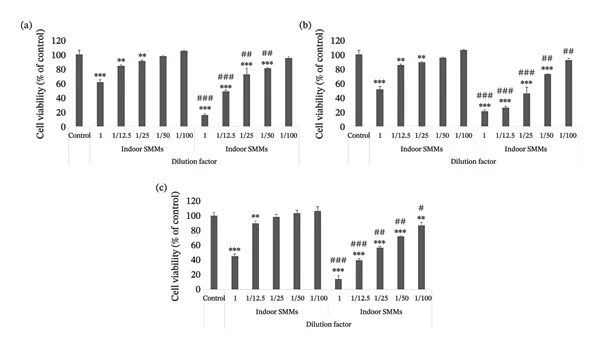
Dose‐dependent cytotoxic effects of SMMs on primary sebocytes and normal human keratinocytes. (a) Cell viability of primary sebocytes after 4 h of exposure to indoor and outdoor SMMs. Cell viability of (b) sebocytes and (c) keratinocytes after 24 h of exposure under conditions identical to those in (a). Cells were treated with serial dilutions (1, 1:12.5, 1:25, 1:50, and 1:100) of indoor and outdoor SMMs formulated based on the metal profiles detected in sebum samples. Cell viability was assessed using the CCK‐8 assay and normalized to that of the untreated control (100%). Data were analyzed using one‐way ANOVA followed by Tukey’s HSD post hoc test; ^∗^ *p* < 0.05, ^∗∗^ *p* < 0.01, ^∗∗∗^ *p* < 0.001 vs. control; ^#^ *p* < 0.05, ^##^ *p* < 0.01, ^###^ *p* < 0.001 vs. indoor SMMs at the same dilution.

To evaluate the cytotoxic effects of SMMs, primary sebocytes and keratinocytes were treated with varying dilutions of indoor and outdoor SMMs for 4 and 24 h (Figure 2a–c). In sebocytes, undiluted (1×) outdoor SMMs exposure decreased cell viability to 15.7 ± 1.7% at 4 h and 21.1 ± 5.1% at 24 h, compared to the control (*p* < 0.001). The indoor SMMs reduced viability at 1× concentration to 61.2 ± 4.2% at 4 h and 51.4 ± 4.2% at 24 h (*p* < 0.001). Even at diluted concentrations, outdoor SMMs exhibited higher cytotoxicity than indoor SMMs.

Keratinocytes displayed a similar pattern: treatment with the undiluted outdoor SMMs significantly decreased cell viability to 14.3 ± 5.0%, whereas the indoor SMMs‐treated cells showed 45.1 ± 3.0% viability (both *p* < 0.001). At 1:12.5 dilution, the outdoor SMMs yielded 39.7 ± 2.3% viability in keratinocytes (*p* < 0.001), compared with 89.8 ± 3.1% in the indoor group (*p* < 0.01). These results indicate that SMMs collected from outdoor environments exhibit greater cytotoxicity in keratinocytes and sebocytes than those collected from indoor environments, particularly at higher concentrations and with longer exposure.

### 3.3. SMMs Induce Lipid Peroxidation in Human Sebocytes

**FIGURE 3:**
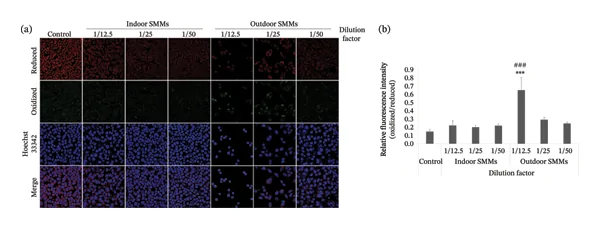
Lipid peroxidation in human sebocytes following exposure to SMMs. (a) Fluorescence microscopy images of human sebocytes stained with BODIPY 581/591 C11 after 24 h of exposure to serial dilutions (1:12.5, 1:25, and 1:50) of indoor and outdoor SMMs. Lipid peroxidation was visualized by the shift in BODIPY C11 fluorescence from red (nonoxidized form) to green (oxidized form). Images are representative of three independent experiments. (b) The fluorescence intensity of lipid peroxidation (oxidized/reduced BODIPY C11 ratio) was calculated using Zen software. Data were analyzed using one‐way ANOVA followed by Tukey’s HSD post hoc test; ^∗∗∗^ *p* < 0.001 vs. control; ^###^ *p* < 0.001 vs. indoor SMMs at the same dilution. Original magnification: 400×. Scale bars: 50 μm.

To investigate the oxidative potential of SMMs, human sebocytes were exposed to various dilutions of indoor and outdoor SMMs, and lipid peroxidation was assessed (Figure 3). Fluorescence microscopy revealed a concentration‐dependent increase in oxidized cells treated with outdoor SMMs, indicating elevated lipid peroxidation. Notably, the 1:25 and 1:50 dilutions markedly increased oxidative stress (Figure 3a). In contrast, sebocytes treated with indoor SMMs exhibited comparatively lower oxidative activity, as supported by the quantitative analysis of the oxidized‐to‐reduced BODIPY C11 fluorescence ratio (Figure 3b). These results indicate that SMMs derived from outdoor worker sebum possess a greater oxidative potential, inducing more pronounced lipid peroxidation in human sebocytes than indoor SMMs.

### 3.4. SMMs in Human Sebocytes Induce DNA Damage

**FIGURE 4:**
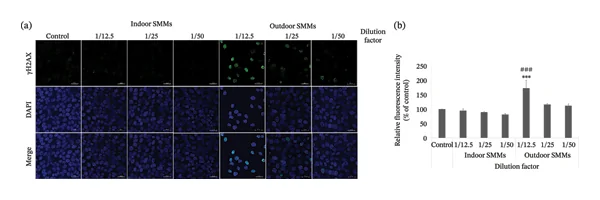
DNA damage response in human sebocytes following exposure to SMMs. (a) Immunofluorescence images of human sebocytes stained for γ‐H2AX after 24 h exposure to serial dilutions (1:12.5, 1:25, and 1:50) of indoor and outdoor SMMs. (b) The fluorescence intensity of γ‐H2AX was calculated using Zen software and normalized to the DAPI fluorescence intensity. Data were analyzed using one‐way ANOVA followed by Tukey’s HSD post hoc test; ^∗^ *p* < 0.05, ^∗∗^ *p* < 0.01, ^∗∗∗^ *p* < 0.001 vs. control; ^###^ *p* < 0.001 vs. indoor SMMs at the same dilution (*n* = 3). Original magnification: 400×. Scale bars: 20 μm.

To evaluate the genotoxic potential of SMMs, γ‐H2AX immunofluorescence staining was performed on human sebocytes following exposure to SMMs (Figure 4a,b). Sebocytes exposed to outdoor SMMs showed a clear, concentration‐dependent increase in γ‐H2AX foci, indicating more DNA double‐strand breaks and activated damage response pathways. Specifically, the 1:12.5 (172.8 ± 27.9% of control, *p* < 0.001), 1:25 (115.9 ± 3.4%), and 1:50 (111.8 ± 6.5%) dilutions significantly increased the γ‐H2AX signal intensity. In contrast, exposure to indoor SMMs did not alter or decrease the γ‐H2AX signal relative to that in the control group. Collectively, these results indicate that outdoor SMMs induce significant genotoxic effects in human sebocytes, whereas indoor SMMs cause comparatively low or no DNA‐damaging activity.

### 3.5. Outdoor SMMs Exacerbate Inflammation and Reduce Differentiation Markers in Human Keratinocytes

**FIGURE 5:**
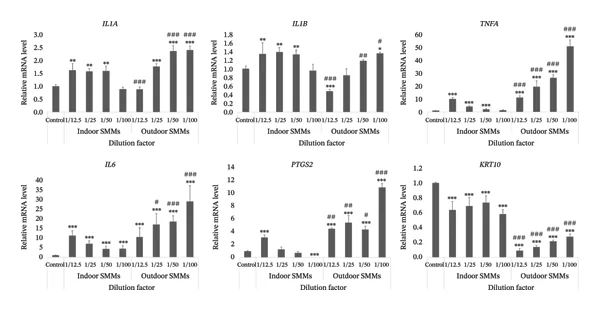
Gene expression analysis of inflammatory and differentiation‐related markers in keratinocytes treated with diluted indoor and outdoor SMMs. Keratinocytes were treated with diluted SMMs (1:12.5, 1:25, 1:50, and 1:100) for 24 h. Relative mRNA expression levels of proinflammatory cytokines (*TNFΑ, IL1Α, IL1Β,* and *IL6*), *PTGS2*, and *KRT10* were assessed using quantitative RT‐PCR. Expression levels were normalized to *RPL13A* and are presented as fold changes relative to the untreated control. Data were analyzed using one‐way ANOVA followed by Tukey’s HSD post hoc test; ^∗^ *p* < 0.05, ^∗∗^ *p* < 0.01, ^∗∗∗^ *p* < 0.001 vs. control; ^#^ *p* < 0.05, ^##^ *p* < 0.01, ^###^ *p* < 0.001 vs. indoor SMMs at the same dilution (*n* = 3).

To assess the proinflammatory and differentiation‐related transcriptional responses of keratinocytes to sebum‐based SMMs, the mRNA expression levels of proinflammatory cytokines (*TNFA, IL1A, IL1B,* and *IL6*), an inflammatory enzyme (*PTGS2)*, and a differentiation marker (*KRT10*) were quantified after 24 h of exposure to indoor and outdoor SMMs (Figure 5). *IL6* expression increased to approximately 29 times that of the control at the 1:100 dilution (*p* < 0.001), whereas *TNFA* expression exceeded a 51‐fold increase at the same dilution (*p* < 0.001). *IL1A* expression levels were also significantly upregulated at intermediate dilution. Cells treated with indoor SMMs exhibited a comparatively modest induction of inflammatory markers; significant elevations in *IL1A, IL1B, IL6,* and *TNFA* were detected at higher concentrations (1:12.5 and 1:25), with expression levels approaching baseline at lower concentrations.

*PTGS2* was also induced by outdoor SMMs at nearly 11‐fold at 1:100 dilution (*p* < 0.001), while indoor SMMs produced milder increases (2–3‐fold at 1:12.5). *KRT10* expression was reduced by indoor and outdoor SMMs, with the outdoor group exhibiting greater suppression across all dilutions (*p* < 0.001). These results suggest that SMMs from outdoor occupational settings induce stronger and broader transcriptional activation of inflammatory pathways while concurrently downregulating differentiation signals in human keratinocytes.

### 3.6. SMMs Activate NF‐κB and p38 MAPK Pathways, Inducing DNA Damage

**FIGURE 6:**
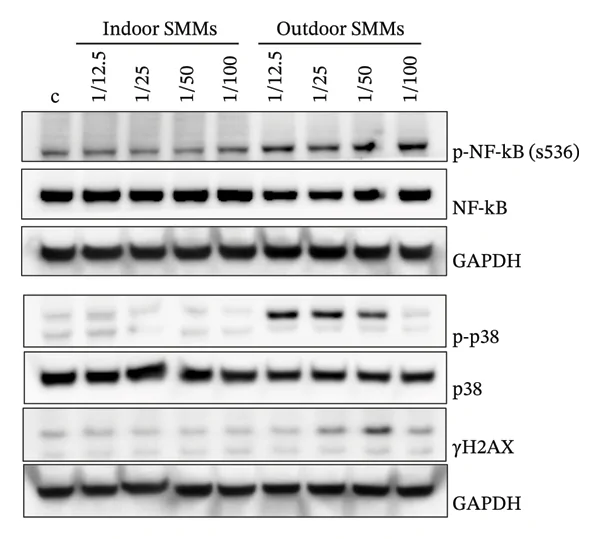
Western blot analysis of NF‐κB, p38 MAPK, and γH2AX in keratinocytes treated with indoor and outdoor SMMs. Keratinocytes were treated with serial dilutions (1:12.5, 1:25, 1:50, and 1:100) of indoor and outdoor SMMs for 24 h. Western blotting was used to analyze the expression of phosphorylated NF‐κB (p‐NF‐κB Ser536), total NF‐κB, phosphorylated p38 (p‐p38), total p38, and the DNA damage marker γH2AX. GAPDH was used as a loading control. C denotes the untreated control.

To elucidate the molecular mechanisms underlying inflammation and DNA damage induced by SMMs, we examined the activation of NF‐κB and p38 MAPK pathways, as well as the expression of the DNA damage marker γH2AX, in human keratinocytes following 24‐h exposure to indoor and outdoor SMMs. Western blot analysis revealed a substantial increase in phosphorylated NF‐κB (pNF‐κB Ser536) levels in keratinocytes treated with outdoor SMMs, particularly at 1:12.5, 1:25, and 1:50 dilutions, whereas no significant changes were observed in cells exposed to indoor SMMs. Similarly, phosphorylated p38 (p‐p38) levels were markedly elevated in keratinocytes exposed to outdoor SMMs, with the highest induction observed at 1:12.5 and 1:25 dilutions. In contrast, cells treated with indoor SMMs exhibited weak or negligible p‐p38 signals. Total p38 levels were similar across all samples, suggesting p38 MAPK pathway activation rather than differences in protein expression (Figure 6). Consistent with the immunofluorescence observations (Figure 4), the levels of γH2AX increased in keratinocytes treated with outdoor SMMs. Overall, these findings indicate that outdoor SMMs from outdoor occupational environments activate NF‐κB and p38 MAPK signaling and induce DNA damage responses in human keratinocytes, whereas indoor SMMs have a less pronounced effect.

### 3.7. Ex Vivo Human Scalp Skin Exposed to Outdoor SMMs Exhibits Increased Inflammation and Aberrant Histological Features

**FIGURE 7:**
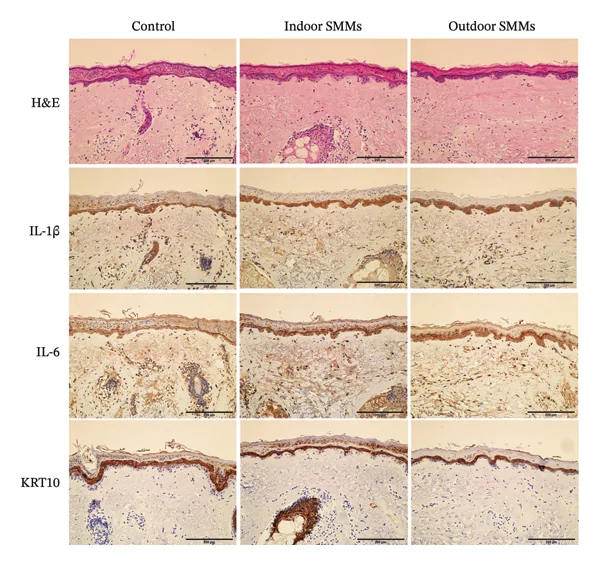
Histological analysis of ex vivo human scalp skin. Human scalp skin was treated with 1:12.5 dilutions of indoor and outdoor SMMs for 6 days. Human scalp skin sections were stained with H&E and antibodies for IL‐1β, IL‐6, and KRT10. Original magnification: 200×. Scale bars: 200 µm.

To assess the physiological impact of SMMs on skin structure and inflammation, ex vivo human skin tissues were treated with 1:12.5 dilutions. Subsequent immunohistochemical analysis revealed that treatment with outdoor or indoor SMMs markedly increased the abundance of IL‐1β and IL‐6 within the epidermal layers in a concentration‐dependent manner. These proinflammatory cytokines were predominantly localized in suprabasal keratinocytes, indicating substantial local inflammatory activation (Figure 7). Conversely, KRT10 expression was notably reduced after exposure to outdoor SMMs, implying impaired epidermal differentiation in response to environmental metal stress (Figure 7).

Hematoxylin and eosin (H&E) staining revealed distinct histopathological differences between the SMMs treatment groups. Tissues treated with outdoor SMMs exhibited disrupted epidermal architecture, including epidermal thinning, irregular basal layer organization, and impaired keratinocyte differentiation, with these changes being more pronounced at the 1:12.5 dilution. In comparison, tissues exposed to indoor SMMs largely retained normal histological features, similar to the untreated controls (Figure 7). Collectively, these findings indicate that even at low concentrations, SMMs reflecting outdoor occupational exposure can provoke substantial inflammatory signaling and structural disruption in human skin, underscoring the significant dermatotoxic potential associated with environmental metal exposure.

## 4. Discussion

This study provides clear evidence that SMMs found in human sebum, particularly those originating from outdoor occupational environments, exert intense cytotoxic, oxidative, proinflammatory, and genotoxic effects on skin‐relevant cells and tissues. By directly extracting metals from the sebum of indoor and outdoor workers, we developed a real‐world exposure model that links environmental metal contamination to biological outcomes in keratinocytes, sebocytes, and ex vivo human skin.

Sebum samples from outdoor workers showed significantly higher concentrations of toxic metals, such as Co, As, Ba, Pb, and Cr, than those from indoor workers. This increase in toxic metal levels is directly correlated with greater cytotoxicity, as evidenced by lower cell viability in keratinocytes and sebocytes. The effects depended on the metal concentration and exposure time, with outdoor SMMs inducing more rapid declines in cell survival, even at lower concentrations, highlighting their enhanced dermatotoxic potential.

Oxidative stress was identified as a central mediator of metal‐induced skin toxicity [32]. Sebocytes exposed to outdoor SMMs exhibited marked lipid peroxidation, as indicated by increased oxidized BODIPY fluorescence intensity. Transition metals such as Fe, Cu, and Mn facilitate redox cycling and generate hydroxyl radicals via Fenton‐type reactions, leading to an overproduction of ROS [33, 34]. This oxidative stress damages lipid membrane integrity, disrupts cellular homeostasis, and contributes to downstream apoptotic signaling [32]. The concurrent induction of γH2AX foci in the current study strongly suggests that oxidative stress drives DNA double‐strand breaks [35]. Consistently, sebocytes and keratinocytes treated with outdoor SMMs showed increased γH2AX expression, highlighting the link between oxidative imbalance and genotoxic stress [35].

Exposure to SMMs also significantly increased the inflammatory signaling. Keratinocytes exposed to outdoor SMMs showed marked upregulation of mRNAs encoding proinflammatory cytokines (*TNFA, IL1A, IL1B,* and *IL6*) and PTGS2. Notably, *TNFA* and *IL-6* expression increased by more than 30–50‐fold, even at low concentrations, emphasizing the potent inflammatory potential of these environmental exposures. Western blot analysis further confirmed that outdoor SMMs strongly activated NF‐κB and p38 MAPK pathways, which are central regulators of stress and inflammatory responses [36–38].

These pathways likely act in concert with AP‐1 transcriptional activity to amplify cytokine release, thereby perpetuating cutaneous inflammation [39]. Ex vivo skin data supported these findings, showing increased IL‐1β and IL‐6 expression in suprabasal keratinocytes.

Exposure to indoor and outdoor SMMs impairs epidermal differentiation. Although both indoor and outdoor SMMs suppressed KRT10 expression, the effect was substantially greater in the outdoor group. Reduced KRT10 disrupts keratinocyte differentiation and weakens the epidermal barrier, potentially contributing to increased transepidermal water loss, increased sensitivity to irritants, and heightened susceptibility to chronic skin disorders, such as atopic dermatitis or acne [40–43]. Consistently, histological examination revealed disrupted epidermal organization and basal layer irregularities following exposure to outdoor SMMs, while indoor SMMs‐treated tissues remained comparatively intact. These results suggest that environmental metals not only induce inflammation and DNA damage but also compromise the structural and functional integrity of the skin barrier.

The findings of this study are consistent with those of previous studies, showing that PM and associated metals activate oxidative and inflammatory signaling in skin cells, often through ROS‐mediated activation of the NF‐κB and MAPK pathways [44, 45]. However, our study directly links real‐world metal accumulation in sebum to specific dermatotoxic outcomes, providing a physiologically relevant model for occupational dermal exposure. Sebum may act as a reservoir for lipophilic pollutants and metals, prolonging their contact with epidermal tissues and creating localized areas of oxidative and inflammatory stress. The role of sebum as a vector for pollutant bioavailability is crucial for understanding the chronic impact of environmental exposure on skin health.

This study has some limitations. Despite significant group differences (*p* < 0.01), the sample size (*n* = 102) and single geographic location (Shanghai) may limit broad generalization. Additionally, while external contamination was strictly controlled, intrinsic factors like diet were not; however, sebum analysis inherently captures the cumulative “total exposome.” Moreover, we used aqueous ionic metal salts to formulate the SMMs. Although heavy metals in sebum likely exist primarily as lipid‐bound organic complexes on the skin surface, the use of water‐soluble metal salts is methodologically essential. Standard submerged in vitro cultures are inherently aqueous; direct application of raw sebum or highly lipophilic complexes induces severe phase separation and cellular hypoxia, which confounds specific metal toxicity. Furthermore, our ICP‐MS analysis quantified the total accumulated metal burden, regardless of the original chemical speciation. The use of highly soluble metal salts allowed us to precisely and reproducibly deliver the exact quantified toxicological burden to the cells. Biologically, once metals penetrate the stratum corneum, they interact with an aqueous interstitial microenvironment. Recent models of dermal bioaccessibility also support the partitioning of environmental metals into bioavailable ionic forms in skin fluids [46]. Consequently, in this aqueous phase, ionic metals represent the active form driving oxidative stress, which aligns with the downstream pathways observed in our previous study [47]. Therefore, future multicenter longitudinal studies are needed to validate these findings across diverse populations.

## 5. Conclusions and Implications

Sebaceous SMMs from outdoor occupational environments exert markedly stronger cytotoxic, oxidative, inflammatory, and genotoxic effects than those from indoor environments. A key contribution of this study is the establishment of a paradigm shift in understanding the role of skin surface lipids: in highly polluted environments, sebum ceases to be merely a protective barrier and instead acts as a “toxic reservoir” that traps and continuously leaches airborne heavy metals into the pores and adjacent tissues. Therefore, this study delivers a critical public health and dermatological message for populations residing in metropolitan areas with high PM levels: proactive pore hygiene and regular, effective removal of contaminated sebum are paramount for preventing pollution‐induced skin aging, barrier dysfunction, and inflammatory diseases. While traditional interventions, such as barrier‐repair agents (e.g., ceramides, provitamin B5) and antioxidant supplementation (e.g., vitamins C and E), remain beneficial, our findings underscore the absolute necessity of advanced deep‐cleansing strategies and topical metal chelators (e.g., EDTA, phytic acid) designed to neutralize and extract these trapped metals directly from the sebaceous environment. In conclusion, addressing sebum‐associated metal exposure can markedly improve the dermatological health of vulnerable occupational groups. Future research should focus on two main directions: (1) longitudinal epidemiological studies to assess the chronic dermatological health risks associated with low‐level, continuous heavy metal exposure through hair follicles and (2) the development of targeted antipollution skincare formulations optimized for pore decontamination and inhibition of metal‐induced signaling pathways in sebocytes.

## Author Contributions

Hye‐Won Na: conceptualization, formal analysis, investigation, validation, writing–original draft, writing–review and editing. Nari Cha: formal analysis, investigation, validation, writing–review and editing. Eun Joo Kim: formal analysis, investigation, validation. Sanghun Kim: formal analysis, investigation, validation. Won‐Seok Park: funding acquisition, investigation, resources. Hyoung‐June Kim: conceptualization, formal analysis, funding acquisition, investigation, resources, writing–original draft, writing–review and editing.

## Funding

This study was supported by the Korea Health Industry Development Institute, RS2023‐KH141286.

## Ethics Statement

This study was conducted in accordance with the Declaration of Helsinki. This study was approved by the Institutional Review Board of the Beijing Sino‐German Union Cosmetic Institute Co, Chinese Academy of Inspection and Quarantine, Beijing, China (IRB approval no. S2019‐01‐002). Written informed consent was obtained from all participants.

## Conflicts of Interest

The authors declare no conflicts of interest.

## Data Availability

The datasets generated and/or analyzed during the current study are available from the corresponding author upon reasonable request.
